# Assessment of Trends in Cigarette Smoking Cessation After Cancer Diagnosis Among US Adults, 2000 to 2017

**DOI:** 10.1001/jamanetworkopen.2020.12164

**Published:** 2020-08-03

**Authors:** Rajesh Talluri, Joël Fokom Domgue, Ellen R. Gritz, Sanjay Shete

**Affiliations:** 1 Department of Data Science, The University of Mississippi Medical Center, Jackson; 2 Department of Epidemiology, The University of Texas MD Anderson Cancer Center, Houston; 3 Division of Cancer Prevention and Population Sciences, The University of Texas MD Anderson Cancer Center, Houston; 4 Department of Gynecologic Oncology and Reproductive Medicine, The University of Texas MD Anderson Cancer Center, Houston; 5 Department of Behavioral Science, The University of Texas MD Anderson Cancer Center, Houston; 6 Department of Biostatistics, The University of Texas MD Anderson Cancer Center, Houston

## Abstract

**Importance:**

Continued cigarette smoking after cancer diagnosis has been shown to adversely alter the cancer survivor’s health outcomes and quality of life. Although considerable progress has been made in reducing cigarette smoking in the United States over the last decades, trends in cigarette smoking cessation among cancer survivors have not yet been fully investigated.

**Objective:**

To evaluate temporal trends in cigarette smoking cessation and their sociodemographic and behavioral correlates in the US population of adult cancer survivors.

**Design, Setting, and Participants:**

This investigation was a serial cross-sectional study of the National Health Interview Survey (NHIS) from 2006 to 2018, a household survey of civilian US residents who were 18 years or older. Data analysis was performed from June to October 2019. The NHIS is population based and representative of the US population. Included in this study were 381 989 respondents to the NHIS in 2006 to 2018.

**Main Outcomes and Measures:**

The primary outcome was the probability of quitting cigarette smoking after first cancer diagnosis. Secondary outcomes were factors associated with quitting cigarette smoking after cancer diagnosis.

**Results:**

Data on 381 989 adults (weighted N = 239 114 051; mean [SD] age, 48.96 [18.28] years; 211 508 [55.37%] female; 61.90% non-Hispanic White, 13.97% non-Hispanic Black, and 16.22% Hispanic individuals) 18 years or older were analyzed from the 2006 to 2018 NHIS, of whom 8.80% (n = 35 524; weighted n = 21 016 720) were diagnosed with cancer. Among cancer survivors diagnosed between 2000 and 2017, the age-adjusted prevalence of current cigarette smoking at the time of first cancer diagnosis was 24.45% (n = 4054; weighted n = 2 395 173). The probability of reporting a cigarette smoking cessation event after first cancer diagnosis statistically significantly increased with each year of cancer diagnosis (hazard ratio [HR], 1.05; 95% CI, 1.02-1.08), indicating upward trends in the prevalence of quitting cigarette smoking over time. Older individuals (HR, 1.02; 95% CI, 1.01-1.03), individuals diagnosed as having a smoking-related cancer (HR, 1.28; 95% CI, 1.06-1.54), individuals with an undergraduate degree (HR, 1.39; 95% CI, 1.08-1.79) or a postgraduate degree (HR, 1.61; 95% CI, 1.18-2.20), and individuals with obesity (HR, 1.32; 95% CI, 1.06-1.63) had a higher probability of reporting a cigarette smoking cessation event after cancer diagnosis, whereas individuals living below the poverty level (HR, 0.62; 95% CI, 0.48-0.81) had a lower probability of reporting a cigarette smoking cessation event after cancer diagnosis.

**Conclusions and Relevance:**

In this nationally representative survey of the US adult population, the likelihood of cigarette smoking cessation among cancer survivors increased with the year of cancer diagnosis from 2000 to 2017; however, the improvement is incremental, and the prevalence of smoking remained high among this population. Considering the projected increase in the population of cancer survivors in the United States, urgent action is needed to increase cigarette smoking cessation rates in this high-risk population.

## Key Points

**Question:**

What were trends in cigarette smoking cessation rates among US adult cancer survivors from 2000 to 2017?

**Findings:**

In this serial cross-sectional study that included 381 989 adults, the age-adjusted prevalence of current cigarette smokers at the time of first cancer diagnosis was 24.25% among cancer survivors diagnosed between 2000-2017. Over the study period, the probability of quitting cigarette smoking after cancer diagnosis statistically significantly increased with the year of cancer diagnosis.

**Meaning:**

In the US adult population of cancer survivors, upward trends in cigarette smoking cessation were observed from 2000 to 2017; however, the prevalence of smoking remained high among this population.

## Teaser

This serial cross-sectional study of the National Health Interview Survey evaluates temporal trends in and sociodemographic and behavioral factors associated with cigarette smoking cessation among adult cancer survivors in the US.

## Introduction

The cancer burden in the United States is substantial, with more than 1.6 million new cases of cancer and approximately 600 000 cancer deaths occurring each year.^1^ In 2019, the United States had more than 16.6 million cancer survivors, a number projected to increase by 2030 to 22.1 million.^1^ As more people diagnosed with cancer are living longer in the United States, the implications of lifestyle behaviors, such as cigarette smoking, that worsen cancer prognosis are of major importance for public health. It is well established that quitting cigarette smoking after cancer diagnosis is associated with better treatment outcomes, improved quality of life, lower risk of second primary cancers, decreased recurrence, and increased survival.^2^ Because cancer diagnosis is a difficult event in people’s lives, it provides an opportunity for clinicians to help patients with cancer reevaluate their lifestyle choices.^3,4^ Considerable progress has been made in reducing cigarette smoking in the United States, and the proportion of smokers who reported receiving physician advice to quit has increased over the last decades.^5^ However, the prevalence of cigarette smoking among cancer survivors remains high.^2,6,7^ A recent cross-sectional analysis of the 2017 National Health Interview Survey (NHIS) data reported that 13.2% of US adult cancer survivors were current smokers,^7^ but trends in cigarette smoking cessation among cancer survivors have not yet been fully investigated. Because the pattern of cigarette smoking varies substantially according to certain sociodemographic factors, such as sex, race/ethnicity, age, educational level, income, and US Census region, comprehensive temporal trend analysis accounting for these factors and using nationally representative data would be essential for the application of targeted cigarette smoking cessation public health programs.

Using the NHIS data, we investigated trends in cigarette smoking cessation rates over time among cancer survivors. In addition, we studied factors associated with quitting cigarette smoking after cancer diagnosis in this high-risk population.

## Methods

### Data and Sampling Design

This serial cross-sectional study was conducted across yearly cycles of the NHIS, a nationally representative household survey designed to monitor the health outcomes in the US population. The NHIS is a cross-sectional household interview survey using a face-to-face interview format, which is targeted to the civilian noninstitutionalized population residing in the United States. The NHIS uses a multistage stratified area probability design to recruit households into the study to perform a representative sampling of the target population. A detailed description of the NHIS sampling design is provided elsewhere.^8^

For this study, the 2006 to 2018 cycles of the NHIS were used. This period was selected because the NHIS sampling method was redesigned as of 2006 to improve the reliability of statistics for racial/ethnic, economic, and geographic domains.^8^ Major changes in the 2006 redesign included state-level stratification in the sampling design, use of an all-area sampling frame, and oversampling of Black, Hispanic, and Asian individuals. Therefore, to maintain consistency, the NHIS data starting from 2006 were used to yield more accurate trend analysis. The 2018 NHIS data were the most recent findings released at the time of our data analyses. Although the selected NHIS cycles included patients with cancer diagnosed as early as the 1970s, our study aimed to assess trends in cigarette smoking cessation in the last 2 decades. Therefore, only individuals who were diagnosed with cancer beginning in 2000 were included.

This study followed the Strengthening the Reporting of Observational Studies in Epidemiology (STROBE) reporting guideline. Respondents to the 2006 to 2018 NHIS cycles gave written informed consent before their participation in the surveys. The Research Ethics Review Board of the National Center for Health Statistics approved the NHIS cycles used in this study. Because the NHIS data were deidentified and publicly available, our secondary data analysis was exempt from institutional review board approval.^9^ Data analysis was performed from June to October 2019.

### Variables and Measures

#### Definition of Cancer Survivor

Cancer survivors in this study were defined using the following definition by the National Cancer Institute: “A person is considered to be a survivor from time of diagnosis of cancer until end of life.”^10^ Therefore, survey respondents who answered yes to the question “Were you ever told by a doctor you have cancer?” were considered cancer survivors in this study. Cancers were classified according to the Centers for Disease Control and Prevention guidelines as smoking-related cancers (SRCs) and non-SRCs (NSRCs).^11^

#### Primary Outcome

Smoking status was defined using the standard NHIS definition of cigarette smoking.^12^ Our primary outcome was the probability of quitting cigarette smoking after first cancer diagnosis. Individuals who were smoking at the time of cancer diagnosis and who reported having quit smoking at a later date were considered to have achieved cigarette smoking cessation after cancer diagnosis. To define the primary outcome, the following 2 metrics were used: age at first cancer diagnosis and age at quitting cigarette smoking. Age at first cancer diagnosis was assessed using the NHIS question “How old were you when this cancer was first diagnosed?” Age at quitting cigarette smoking was computed using the variable time since quit smoking (in years). In patients with multiple cancers, smoking status at the onset of the first cancer was used. Therefore, cancer survivors whose age at first cancer diagnosis was younger than their age at quitting cigarette smoking and who started to smoke before a diagnosis of cancer were considered to have quit smoking after cancer diagnosis and classified as being current smokers at the time of cancer diagnosis. Those whose age at first cancer diagnosis was older than their age at quitting cigarette smoking were considered to have quit smoking before cancer diagnosis and were excluded from this study. Individuals who reported first cancer diagnosis the same year as the year when they quit smoking were also excluded from our analysis because it was not possible to determine the sequence of occurrence of these 2 events (cancer diagnosis and quitting cigarette smoking).

#### Covariates

Covariates used in this study included the sociodemographic and behavioral characteristics known to be associated with smoking behavior in the US population.^2^ These factors were sex, race/ethnicity, age, educational level, poverty level, body mass index (BMI) (calculated as weight in kilograms divided by height in meters squared), alcohol consumption, and US Census region. Because our main objective was to evaluate temporal trends in cigarette smoking cessation among adult cancer survivors, the year of first cancer diagnosis and the year of NHIS administration were also included. Respondents’ age (in years) at the time of NHIS administration, the year of first cancer diagnosis (2000 to 2017), and the year each NHIS cycle was administered (2006 to 2018) were included as continuous variables in our analysis.

### Statistical Analysis

Data from the adult section of the 2006 to 2018 NHIS cycles (ie, civilian US residents who were 18 years or older) were used in this analysis. The multistage sampling approach of the NHIS made it possible to create a design weight for each survey respondent, which conveys the number of population units each NHIS respondent represents. These design weights were computed as the product of inverses of the probability of selection at each individual stage of the survey based on the sampling design. The design weights were then adjusted for nonresponse and further adjusted using poststratification by sex, race/ethnicity, and age based on population estimates from the recent US Census information at the time of each NHIS administration. Weights were computed and provided by the NHIS for each cycle from 2006 to 2018.

During that interval, 2 different sampling designs were used by the NHIS, one from 2006 to 2015 and another from 2016 to 2018. The changes in the sampling design made in 2016 included updating sampling areas based on the changes in distribution of the US population since the previous design in 2006 and removal of oversampling procedures for Black, Hispanic, and Asian individuals.^13^ Considering these 2 different sampling designs, combining the NHIS data from 2006 to 2018 required additional adjustment of weights per NHIS cycle so that the addition of all weights accurately represents the survey population. We performed this weight adjustment per the recommendations of the NHIS description report.^14^ Briefly, we first adjusted the weights for each sampling design period (2006-2015 and 2016-2018) separately. We then performed a second level of weight adjustment of the data from the 2 study periods (2006-2015 and 2016-2018) to derive the final weights that were consistent for the entire study period (2006-2018). Age-adjusted estimates were calculated by stratifying the survey data into 3 age categories (18-44, 45-64, and ≥65 years) and compared with the 2010 US Census estimates for these age categories. We used the R package “survey”^15^ to calculate estimated weight-adjusted prevalence.

Study participants were categorized based on the year in which they were diagnosed with cancer (2000-2017). For each NHIS cycle, individuals who reported their first cancer diagnosis to be the same year as the NHIS were excluded from this analysis because it was not possible to determine their smoking behavior after cancer diagnosis. The sociodemographic and behavioral characteristics of cancer survivors who were current smokers at the time of diagnosis for each year of cancer diagnosis from 2000 to 2017 were described.

To assess trends and identify factors associated with quitting cigarette smoking in individuals who were current smokers at the time of cancer diagnosis, a survey Cox proportional hazards model^16^ was used to model the time to quit after cancer diagnosis based on several variables and to show the resulting survival curves. The year of first cancer diagnosis was included in the model to test for any trends in the prevalence of quitting cigarette smoking after cancer diagnosis. The survey Cox proportional hazards model was adjusted for sex, race/ethnicity, age, cancer type (NSRC or SRC), educational level, poverty level, BMI, alcohol consumption, and US Census region (Northeast, Midwest, South, or West). In addition, the model for the NHIS cycle (the year the survey was administered) was adjusted to account for any potential bias that could be associated with the technical or logistic improvements in the implementation and administration of the NHIS over the years. Participants with missing data on any covariate included in the model were excluded from the analysis. The validity of the survey Cox proportional hazards model assumption was evaluated using Schoenfeld residual plots.^17^ To facilitate the interpretation of hazard ratios (HRs) computed from the survey Cox proportional hazards model, we showed the resulting survival curves at different years of first cancer diagnosis (2000, 2005, 2010, and 2015). The survival curves show how the event (quitting cigarette smoking) probabilities change by year of cancer diagnosis according to the time (in years) elapsed since first cancer diagnosis.

The statistical analyses were performed using the R package “survey” (version 3.6.1; R Foundation). For all analyses, the level of statistical significance was defined as 2-sided *P* ≤ .05.

## Results

### Characteristics of the Study Population

The 2006 to 2018 NHIS data included 381 989 adults (weighted N = 239 114 051; mean [SD] age, 48.96 [18.28] years; 211 508 [55.37%] female; and 61.90% non-Hispanic White; 13.97% non-Hispanic Black; and 16.22% Hispanic individuals) (weighted N = 239 114 051) from the 2006 to 2018 NHIS who were 18 years or older. Of these individuals, 8.80% (n = 35 524; weighted n = 21 016 720) were diagnosed with cancer.

Among these cancer survivors, 20.33% (n = 7765; weighted n = 4 247 967) were current smokers at the time of cancer diagnosis. Of these individuals, 60.40% (n = 4391; weighted n = 2 565 795) reported first cancer diagnosis in or after 2000. Excluding 337 individuals who reported first cancer diagnosis the same year as NHIS administration, a study sample of 4054 (weighted n = 2 395 173) cancer survivors was included in our analysis.

Table 1 lists the age-adjusted prevalence, and Table 2 lists the sociodemographic characteristics of US adult cancer survivors who were current smokers at the time of cancer diagnosis overall (2000-2017) and by year of cancer diagnosis. Among this population of cancer survivors, the age-adjusted prevalence of current cigarette smokers at the time of first cancer diagnosis was 24.45% (n = 4054; weighted n = 2 395 173). Overall, 53.72% (survey weighted) of cancer survivors who were smoking at the time of cancer diagnosis were female, 83.02% were non-Hispanic White, 7.76% were non-Hispanic Black, and 4.31% were Hispanic. Only 17.59% of this population were aged 18 to 44 years, 48.74% were aged 45 to 64 years, and 33.66% were 65 years or older; 33.79% had an educational level of high school graduate or general equivalency diploma (GED), and 16.65% were living below the poverty level. Cancer type was NSRC in 67.87% and SRC in 32.13%.

**Table 1.** Age-Adjusted Prevalence of US Adult Cancer Survivors Who Were Current Cigarette Smokers at the Time of First Cancer Diagnosis by Year of Cancer Diagnosis From 2000 to 2017

Variable	Total study sample	Year of cancer diagnosis								
		2000	2001	2002	2003	2004	2005	2006	2007	2008
No. of cancer survivors	20 108	1073	1109	1179	1258	1321	1539	1505	1487	1498
Weighted No. of cancer survivors	12 803 888	540 056	600 884	650 622	714 334	673 207	764 200	860 300	799 165	877 590
Age-adjusted % (95% CI) of individuals who were current smokers at diagnosis	24.45 (22.98-25.99)	30.88 (25.03-37.42)	29.93 (24.09-36.50)	31.28 (25.49-37.72)	29.36 (23.93-35.44)	28.00 (22.22-34.61)	29.40 (24.59-34.72)	27.91 (23.28-33.07)	25.48 (20.04-31.80)	24.80 (19.78-30.60)
		**2009**	**2010**	**2011**	**2012**	**2013**	**2014**	**2015**	**2016**	**2017**
No. of cancer survivors	20 108	1397	1349	1224	1120	955	816	594	471	213
Weighted No. of cancer survivors	12 803 888	749 194	796 473	783 307	810 416	733 969	767 274	726 376	667 265	289 256
Age-adjusted % (95% CI) of individuals who were current smokers at diagnosis	24.45 (22.98-25.99)	21.42 (16.59-27.19)	19.12 (14.40-24.95)	22.85 (16.03-31.47)	21.78 (15.97-28.98)	18.28 (13.79-23.83)	23.68 (17.67-30.98)	21.11 (15.27-28.44)	20.49 (14.67-27.87)	16.11 (9.68-25.59)

**Table 2.** Sociodemographic Characteristics of US Adult Cancer Survivors Who Were Current Cigarette Smokers at the Time of First Cancer Diagnosis by Year of Cancer Diagnosis From 2000 to 2017

Variable	Total study sample			Weighted % (95% CI) by year of cancer diagnosis								
	Raw No.	Weighted No.	Weighted % (95% CI)	2000	2001	2002	2003	2004	2005	2006	2007	2008
Sex												
Female	2277	1 286 799	53.72 (51.43-56.00)	62.86 (54.59-70.44)	64.89 (55.63-73.16)	65.04 (57.07-72.25)	55.34 (46.90-63.49)	53.96 (45.19-62.48)	51.52 (43.87-59.10)	54.62 (46.50-62.50)	59.31 (50.92-67.19)	49.56 (40.95-58.19)
Male	1777	1 108 374	46.28 (44.00-48.57)	37.14 (29.56-45.41)	35.11 (26.84-44.37)	34.96 (27.75-42.93)	44.66 (36.51-53.10)	46.04 (37.52-54.81)	48.48 (40.90-56.13)	45.38 (37.50-53.50)	40.69 (32.81-49.08)	50.44 (41.81-59.05)
Race/ethnicity												
Non-Hispanic White	3228	1 987 251	83.02 (81.24-84.67)	87.05 (80.03-91.85)	82.17 (74.17-88.09)	86.84 (81.39-90.88)	88.12 (83.13-91.78)	85.14 (78.16-90.17)	81.84 (75.62-86.75)	83.16 (76.03-88.49)	83.68 (77.02-88.70)	85.95 (79.34-90.70)
Non-Hispanic Black	419	185 663	7.76 (6.66-9.01)	6.17 (2.98-12.35)	10.51 (6.25-17.16)	6.28 (3.63-10.65)	7.54 (4.62-12.09)	6.41 (3.69-10.93)	6.78 (4.23-10.70)	7.88 (4.77-12.73)	6.03 (3.50-10.21)	6.36 (3.92-10.15)
Hispanic	212	103 072	4.31 (3.44-5.38)	1.73 (0.80-3.68)	1.79 (0.69-4.52)	2.68 (1.20-5.91)	3.14 (1.74-5.59)	2.38 (1.01-5.49)	5.46 (3.02-9.67)	5.78 (2.82-11.47)	7.78 (4.23-13.87)	2.43 (1.27-4.59)
Other	188	117 669	4.92 (3.97-6.08)	5.05 (2.26-10.93)	5.53 (2.33-12.58)	4.20 (2.03-8.47)	1.20 (0.41-3.41)	6.07 (2.77-12.78)	5.92 (3.06-11.16)	3.18 (1.08-9.02)	2.51 (1.10-5.62)	5.26 (2.04-12.94)
Age, y												
18-44	743	421 430	17.59 (15.91-19.42)	20.35 (14.59-27.63)	18.07 (12.16-26.01)	22.70 (16.50-30.38)	20.32 (14.55-27.65)	15.78 (10.44-23.14)	21.74 (16.21-28.52)	17.33 (12.33-23.79)	21.32 (14.69-29.89)	14.40 (9.31-21.62)
45-64	1886	1 167 502	48.74 (46.52-50.97)	41.78 (33.59-50.45)	45.73 (36.58-55.19)	42.29 (34.51-50.46)	41.33 (33.43-49.72)	49.32 (40.73-57.96)	49.86 (42.67-57.06)	48.97 (41.15-56.83)	53.01 (43.70-62.11)	53.58 (44.96-61.99)
≥65	1425	806 240	33.66 (31.65-35.74)	37.88 (29.80-46.69)	36.19 (27.78-45.55)	35.01 (27.92-42.84)	38.34 (30.39-46.98)	34.90 (27.47-43.14)	28.40 (22.38-35.29)	33.71 (26.82-41.36)	25.67 (18.45-34.52)	32.02 (24.28-40.90)
Cancer type												
Non–smoking-related cancer	2678	1 625 723	67.87 (65.67-70.00)	64.62 (56.43-72.03)	72.98 (64.52-80.04)	71.31 (63.38-78.12)	70.00 (62.96-76.21)	68.57 (60.10-75.95)	67.94 (61.14-74.05)	70.35 (62.76-76.96)	74.13 (66.46-80.56)	62.47 (53.71-70.48)
Smoking-related cancer	1376	769 450	32.13 (30.00-34.33)	35.38 (27.97-43.57)	27.02 (19.96-35.48)	28.69 (21.88-36.62)	30.00 (23.79-37.04)	31.43 (24.05-39.90)	32.06 (25.95-38.86)	29.65 (23.04-37.24)	25.87 (19.44-33.54)	37.53 (29.52-46.29)
Educational level												
High school graduate or GED	1348	805 867	33.79 (31.67-35.96)	31.99 (24.97-39.93)	29.24 (22.20-37.42)	32.54 (25.18-40.87)	30.65 (23.52-38.84)	35.43 (27.27-44.54)	36.11 (29.29-43.54)	32.92 (26.04-40.63)	32.49 (25.01-40.97)	30.83 (23.40-39.41)
No high school diploma	704	369 871	15.51 (13.98-17.16)	17.41 (11.22-26.03)	9.47 (5.72-15.30)	16.33 (11.35-22.93)	14.42 (9.71-20.89)	18.93 (13.71-25.54)	12.86 (9.02-18.00)	18.70 (13.45-25.40)	17.41 (12.21-24.22)	11.90 (8.20-16.97)
Some college or AA degree	1324	782 565	32.81 (30.71-34.98)	35.11 (27.57-43.47)	36.57 (28.12-45.94)	33.45 (25.98-41.84)	35.01 (27.14-43.78)	27.17 (20.37-35.23)	35.45 (28.11-43.54)	33.08 (25.38-41.81)	31.74 (24.24-40.33)	38.97 (31.06-47.50)
Undergraduate degree	445	294 264	12.34 (10.87-13.97)	9.27 (5.20-15.97)	18.41 (11.51-28.12)	11.66 (7.76-17.14)	10.94 (6.79-17.15)	13.71 (8.30-21.81)	12.02 (8.16-17.37)	11.47 (7.13-17.93)	11.86 (7.06-19.26)	12.20 (7.32-19.64)
Postgraduate degree	216	132 705	5.56 (4.68-6.60)	6.22 (3.35-11.27)	6.31 (3.25-11.90)	6.03 (3.60-9.93)	8.98 (4.92-15.85)	4.76 (2.58-8.64)	3.56 (1.77-7.04)	3.83 (1.85-7.75)	6.50 (3.49-11.79)	6.10 (3.39-10.74)
Poverty level												
Living above the poverty level	2994	1 890 099	83.35 (81.59-84.97)	80.74 (72.40-87.01)	81.35 (72.89-87.62)	83.70 (77.58-88.40)	86.55 (79.39-91.49)	83.57 (76.37-88.90)	86.18 (81.03-90.10)	84.46 (78.49-89.01)	83.01 (75.35-88.65)	87.26 (82.01-91.14)
Living below the poverty level	816	377 679	16.65 (15.03-18.41)	19.26 (12.99-27.60)	18.65 (12.38-27.11)	16.30 (11.60-22.42)	13.45 (8.51-20.61)	16.43 (11.10-23.63)	13.82 (9.90-18.97)	15.54 (10.99-21.51)	16.99 (11.35-24.65)	12.74 (8.86-17.99)
Body mass index												
Normal weight	1417	824 151	34.41 (32.31-36.57)	36.84 (28.86-45.62)	35.01 (26.84-44.16)	34.10 (26.80-42.24)	31.24 (24.68-38.65)	38.18 (29.72-47.41)	33.64 (26.85-41.18)	31.56 (24.79-39.20)	37.34 (30.11-45.18)	36.28 (28.69-44.61)
Underweight	139	68 653	2.87 (2.24-3.66)	3.30 (1.48-7.20)	2.00 (0.88-4.46)	1.95 (0.82-4.54)	1.33 (0.44-3.96)	3.27 (1.58-6.65)	2.08 (0.93-4.57)	2.05 (0.96-4.35)	2.31 (1.03-5.10)	1.49 (0.58-3.77)
Overweight	1290	792 808	33.10 (30.98-35.29)	29.13 (22.40-36.93)	31.72 (23.53-41.22)	36.71 (29.29-44.81)	36.42 (27.87-45.92)	28.98 (22.19-36.86)	34.38 (27.93-41.46)	35.47 (28.15-43.54)	27.51 (19.96-36.59)	28.67 (21.85-36.62)
Obese	1208	709 562	29.62 (27.52-31.82)	30.73 (23.49-39.06)	31.27 (23.22-40.64)	27.25 (20.83-34.77)	31.01 (23.94-39.09)	29.57 (22.26-38.12)	29.90 (23.44-37.28)	30.92 (24.05-38.75)	32.85 (25.47-41.19)	33.56 (25.91-42.19)
Alcohol consumption												
Never drinker	333	181 519	7.65 (6.51-8.96)	6.54 (3.45-12.04)	7.11 (4.00-12.33)	8.91 (5.49-14.13)	9.40 (5.13-16.62)	8.21 (4.39-14.82)	11.34 (7.01-17.82)	6.30 (3.48-11.16)	5.33 (2.96-9.42)	6.29 (3.20-12.00)
Former drinker	1091	616 922	25.99 (24.12-27.95)	33.88 (25.80-43.02)	24.83 (17.72-33.63)	29.03 (22.23-36.91)	23.71 (17.52-31.26)	26.92 (19.72-35.59)	29.24 (22.95-36.44)	21.84 (16.62-28.14)	20.35 (14.62-27.60)	22.85 (17.11-29.83)
Current drinker	2588	1 575 556	66.37 (64.25-68.42)	59.58 (50.48-68.06)	68.06 (59.05-75.89)	62.07 (53.66-69.81)	66.88 (58.50-74.32)	64.87 (55.89-72.91)	59.43 (52.10-66.35)	71.86 (65.00-77.83)	74.32 (66.52-80.82)	70.86 (63.02-77.62)
US Census region												
Northeast	633	371 665	15.52 (13.62-17.62)	17.31 (11.47-25.28)	12.31 (7.57-19.40)	16.48 (10.83-24.28)	18.14 (12.44-25.68)	16.49 (10.55-24.86)	13.50 (8.46-20.86)	16.49 (11.79-22.57)	16.45 (10.99-23.89)	19.26 (13.03-27.52)
Midwest	1017	626 416	26.15 (23.95-28.49)	21.89 (15.42-30.11)	30.28 (22.38-39.54)	23.61 (16.93-31.90)	24.53 (18.30-32.05)	23.86 (17.30-31.94)	27.91 (22.03-34.66)	30.47 (23.42-38.57)	28.39 (20.98-37.17)	27.67 (20.95-35.58)
South	1563	963 674	40.23 (37.71-42.81)	45.13 (36.48-54.07)	38.14 (30.13-46.86)	39.72 (32.58-47.33)	34.36 (26.88-42.71)	42.67 (34.57-51.19)	35.18 (28.64-42.34)	36.41 (29.23-44.26)	40.74 (32.12-49.97)	34.68 (27.12-43.11)
West	841	433 418	18.10 (16.23-20.12)	15.67 (10.88-22.05)	19.27 (12.65-28.24)	20.18 (14.49-27.40)	22.97 (16.14-31.60)	16.98 (11.38-24.57)	23.41 (17.25-30.93)	16.63 (11.93-22.71)	14.42 (9.19-21.91)	18.39 (12.30-26.57)
				**2009**	**2010**	**2011**	**2012**	**2013**	**2014**	**2015**	**2016**	**2017**
Sex												
Female	2277	1 286 799	53.72 (51.43-56.00)	47.13 (37.68-56.80)	54.73 (45.12-64.01)	44.65 (32.97-56.96)	51.83 (41.11-62.39)	51.61 (40.13-62.93)	54.38 (43.59-64.78)	48.01 (36.83-59.39)	45.69 (33.89-58.00)	48.14 (26.80-70.18)
Male	1777	1 108 374	46.28 (44.00-48.57)	52.87 (43.20-62.32)	45.27 (35.99-54.88)	55.35 (43.04-67.03)	48.17 (37.61-58.89)	48.39 (37.07-59.87)	45.62 (35.22-56.41)	51.99 (40.61-63.17)	54.31 (42.00-66.11)	51.86 (29.82-73.20)
Race/ethnicity												
Non-Hispanic White	3228	1 987 251	83.02 (81.24-84.67)	79.85 (68.05-88.06)	86.58 (79.59-91.44)	80.48 (69.97-87.95)	85.27 (77.72-90.57)	84.76 (75.45-90.97)	83.74 (74.43-90.11)	73.96 (62.06-83.14)	77.81 (65.36-86.70)	64.50 (41.44-82.35)
Non-Hispanic Black	419	185 663	7.76 (6.66-9.01)	6.69 (3.12-13.76)	6.51 (3.13-13.06)	8.24 (4.61-14.28)	8.18 (4.26-15.13)	7.48 (3.46-15.42)	9.06 (4.68-16.81)	6.18 (2.66-13.69)	13.14 (6.75-24.03)	18.09 (6.15-42.65)
Hispanic	212	103 072	4.31 (3.44-5.38)	5.90 (1.66-18.87)	4.14 (2.06-8.14)	3.39 (1.10-10.03)	3.16 (1.40-6.96)	2.83 (0.74-10.20)	2.25 (0.80-6.18)	11.02 (5.40-21.16)	7.30 (2.31-20.78)	5.76 (1.20-23.53)
Other	188	117 669	4.92 (3.97-6.08)	7.57 (2.91-18.26)	2.77 (1.22-6.15)	7.89 (3.08-18.77)	3.39 (1.53-7.33)	4.93 (2.13-10.98)	4.95 (2.32-10.25)	8.85 (3.57-20.29)	1.75 (0.52-5.75)	11.65 (2.89-36.90)
Age, y												
18-44	743	421 430	17.59 (15.91-19.42)	13.64 (8.28-21.66)	14.72 (8.66-23.89)	14.42 (7.05-27.23)	20.15 (12.16-31.53)	15.09 (9.19-23.80)	19.56 (11.56-31.15)	14.23 (7.99-24.05)	14.94 (8.20-25.67)	17.09 (5.55-41.94)
45-64	1886	1 167 502	48.74 (46.52-50.97)	53.78 (43.98-63.30)	51.16 (41.27-60.96)	53.78 (41.27-65.83)	45.89 (35.66-56.48)	49.60 (38.10-61.14)	43.43 (33.02-54.45)	54.57 (43.10-65.58)	55.19 (43.14-66.66)	35.47 (17.22-59.23)
≥65	1425	806 240	33.66 (31.65-35.74)	32.58 (24.65-41.65)	34.12 (25.94-43.38)	31.80 (22.37-43.00)	33.95 (25.03-44.19)	35.31 (25.11-47.04)	37.01 (27.71-47.38)	31.20 (21.82-42.43)	29.87 (19.86-42.28)	47.44 (26.43-69.39)
Cancer type												
Non–smoking-related cancer	2678	1 625 723	67.87 (65.67-70.00)	72.00 (63.07-79.48)	63.62 (53.03-73.03)	71.45 (61.33-79.80)	66.80 (56.86-75.43)	62.02 (49.94-72.77)	71.02 (59.99-80.02)	70.40 (58.99-79.72)	54.13 (40.86-66.84)	56.12 (33.87-76.15)
Smoking-related cancer	1376	769 450	32.13 (30.00-34.33)	28.00 (20.52-36.93)	36.38 (26.97-46.97)	28.55 (20.20-38.67)	33.20 (24.57-43.14)	37.98 (27.23-50.06)	28.98 (19.98-40.01)	29.60 (20.28-41.01)	45.87 (33.16-59.14)	43.88 (23.85-66.13)
Educational level												
High school graduate or GED	1348	805 867	33.79 (31.67-35.96)	37.93 (28.62-48.21)	35.01 (26.22-44.96)	32.21 (21.99-44.46)	43.60 (33.73-54.00)	29.50 (19.94-41.29)	40.30 (30.37-51.09)	29.46 (20.36-40.56)	34.68 (23.48-47.88)	30.19 (14.17-53.12)
No high school diploma	704	369 871	15.51 (13.98-17.16)	15.55 (9.30-24.85)	12.82 (7.69-20.61)	12.89 (7.35-21.64)	9.00 (5.48-14.45)	20.34 (12.23-31.86)	10.25 (6.43-15.95)	21.49 (13.10-33.20)	27.12 (17.64-39.26)	14.47 (3.76-42.29)
Some college or AA degree	1324	782 565	32.81 (30.71-34.98)	29.28 (21.56-38.41)	36.31 (27.83-45.74)	33.50 (21.83-47.61)	30.82 (21.17-42.48)	35.45 (24.96-47.55)	31.39 (22.95-41.28)	26.55 (17.71-37.78)	25.98 (16.95-37.65)	32.22 (15.63-54.94)
Undergraduate degree	445	294 264	12.34 (10.87-13.97)	10.91 (6.74-17.20)	7.21 (3.94-12.85)	17.68 (10.55-28.10)	13.55 (7.09-24.37)	6.50 (2.91-13.92)	15.44 (8.36-26.78)	11.46 (6.30-19.95)	11.35 (5.18-23.07)	20.17 (7.72-43.31)
Postgraduate degree	216	132 705	5.56 (4.68-6.60)	6.33 (3.15-12.29)	8.64 (4.39-16.31)	3.73 (1.37-9.72)	3.03 (1.12-7.92)	8.21 (4.31-15.07)	2.61 (0.81-8.10)	11.03 (5.47-21.00)	0.87 (0.12-6.18)	2.94 (0.55-14.25)
Poverty level												
Living above the poverty level	2994	1 890 099	83.35 (81.59-84.97)	79.84 (70.40-86.83)	83.34 (74.73-89.43)	86.66 (78.14-92.19)	79.04 (68.06-86.96)	83.87 (75.70-89.67)	88.20 (80.18-93.25)	80.16 (69.24-87.88)	72.83 (61.04-82.10)	86.07 (62.43-95.83)
Living below the poverty level	816	377 679	16.65 (15.03-18.41)	20.16 (13.17-29.60)	16.66 (10.57-25.27)	13.34 (7.81-21.86)	20.96 (13.04-31.94)	16.13 (10.33-24.30)	11.80 (6.75-19.82)	19.84 (12.12-30.76)	27.17 (17.90-38.96)	13.93 (4.17-37.57)
Body mass index												
Normal weight	1417	824 151	34.41 (32.31-36.57)	32.94 (25.10-41.86)	38.03 (28.86-48.14)	35.60 (25.52-47.15)	33.97 (24.92-44.36)	28.90 (19.67-40.28)	32.03 (23.07-42.54)	27.38 (18.12-39.11)	43.10 (30.41-56.77)	34.64 (17.09-57.66)
Underweight	139	68 653	2.87 (2.24-3.66)	6.01 (1.94-17.12)	0.95 (0.40-2.25)	3.04 (0.98-9.06)	4.93 (2.43-9.75)	3.14 (1.23-7.78)	4.39 (2.01-9.30)	4.20 (1.26-13.10)	4.11 (1.54-10.51)	0.00 (0.00-0.00)
Overweight	1290	792 808	33.10 (30.98-35.29)	28.66 (20.53-38.43)	25.02 (17.71-34.09)	32.64 (20.85-47.12)	36.35 (26.79-47.12)	41.01 (29.81-53.24)	36.71 (27.10-47.50)	41.88 (30.88-53.76)	27.48 (17.47-40.42)	46.09 (25.79-67.78)
Obese	1208	709 562	29.62 (27.52-31.82)	32.39 (23.89-42.24)	36.00 (27.61-45.34)	28.72 (18.68-41.40)	24.75 (16.61-35.21)	26.95 (18.35-37.71)	26.87 (18.59-37.16)	26.53 (17.77-37.64)	25.31 (15.65-38.22)	19.27 (8.81-37.09)
Alcohol consumption												
Never drinker	333	181 519	7.65 (6.51-8.96)	10.09 (4.76-20.15)	7.25 (3.22-15.53)	7.33 (3.72-13.96)	4.98 (2.54-9.54)	11.90 (6.58-20.59)	6.61 (2.62-15.69)	5.67 (2.24-13.63)	6.98 (3.15-14.72)	7.66 (1.20-36.23)
Former drinker	1091	616 922	25.99 (24.12-27.95)	25.95 (18.56-35.01)	29.72 (20.83-40.46)	17.92 (10.83-28.19)	22.39 (15.57-31.09)	32.87 (22.64-45.03)	24.15 (16.79-33.45)	30.66 (20.93-42.47)	33.19 (22.45-46.02)	18.59 (6.40-43.26)
Current drinker	2588	1 575 556	66.37 (64.25-68.42)	63.96 (53.91-72.92)	63.03 (52.41-72.52)	74.75 (63.81-83.24)	72.63 (63.64-80.09)	55.23 (43.64-66.28)	69.24 (59.19-77.74)	63.67 (51.83-74.05)	59.83 (47.13-71.34)	73.75 (48.75-89.24)
US Census region												
Northeast	633	371 665	15.52 (13.62-17.62)	13.29 (8.18-20.87)	13.89 (8.97-20.89)	12.33 (7.46-19.71)	15.56 (8.96-25.67)	18.94 (10.68-31.35)	13.83 (7.70-23.59)	9.42 (4.58-18.38)	18.16 (9.76-31.28)	20.87 (7.68-45.55)
Midwest	1017	626 416	26.15 (23.95-28.49)	25.92 (19.19-34.02)	27.25 (19.95-36.02)	20.97 (13.73-30.66)	29.80 (20.94-40.48)	30.49 (20.87-42.17)	29.17 (20.45-39.75)	21.38 (13.80-31.59)	21.30 (12.46-33.99)	18.04 (5.68-44.60)
South	1563	963 674	40.23 (37.71-42.81)	41.39 (32.39-51.01)	39.34 (29.85-49.70)	42.48 (30.00-55.99)	42.00 (32.18-52.50)	38.99 (28.49-50.61)	43.15 (33.20-53.69)	49.70 (38.39-61.04)	44.12 (31.13-57.97)	40.41 (21.36-62.88)
West	841	433 418	18.10 (16.23-20.12)	19.39 (12.35-29.11)	19.52 (13.22-27.85)	24.22 (15.85-35.18)	12.64 (6.89-22.05)	11.59 (6.62-19.52)	13.84 (8.65-21.43)	19.51 (11.34-31.45)	16.41 (6.80-34.56)	20.67 (8.13-43.43)

Abbreviations: AA, associate degree; GED, general equivalency diploma.

### Trends in Cigarette Smoking Cessation After Cancer Diagnosis

After adjusting for potential confounders, the probability of reporting a cigarette smoking cessation event after first cancer diagnosis statistically significantly increased with each year of cancer diagnosis (HR, 1.05; 95% CI, 1.02-1.08) in the survey Cox proportional hazards model (Table 3). This finding indicated upward trends in the prevalence of quitting cigarette smoking among the population of adult cancer survivors over time in the United States.

**Table 3.** Survey Cox Proportional Hazards Model of the Time to Quit Smoking Among US Adult Cancer Survivors Who Were Current Cigarette Smokers at the Time of First Cancer Diagnosis

Variable	Hazard ratio (95% CI)	*P* value
Year of cancer diagnosis	1.05 (1.02-1.08)	.002
Age	1.02 (1.01-1.03)	<.001
Year of NHIS administration	0.96 (0.93-0.99)	.008
Sex		
Female	1 [Reference]	NA
Male	1.15 (0.95-1.38)	.15
Race/ethnicity		
Non-Hispanic White	1 [Reference]	NA
Non-Hispanic Black	0.94 (0.66-1.33)	.73
Hispanic	1.30 (0.90-1.88)	.17
Other^a^	1.06 (0.68-1.66)	.79
Cancer type		
Non–smoking-related cancer	1 [Reference]	NA
Smoking-related cancer	1.28 (1.06-1.54)	.01
Educational level		
High school graduate or GED	1 [Reference]	NA
No high school diploma	0.89 (0.67-1.19)	.45
Some college or AA degree	1.04 (0.83-1.31)	.71
Undergraduate degree	1.39 (1.08-1.79)	.01
Postgraduate degree	1.61 (1.18-2.20)	.003
Poverty level		
Living above the poverty level	1 [Reference]	NA
Living below the poverty level	0.62 (0.48-0.81)	<.001
Body mass index		
Normal weight	1 [Reference]	NA
Underweight	0.96 (0.47-1.93)	.90
Overweight	1.11 (0.90-1.37)	.34
Obese	1.32 (1.06-1.63)	.01
Alcohol consumption		
Never drinker	1 [Reference]	NA
Former drinker	1.13 (0.78-1.63)	.53
Current drinker	1.11 (0.78-1.57)	.57
US Census region		
Northeast	1 [Reference]	NA
Midwest	0.82 (0.64-1.05)	.12
South	0.81 (0.64-1.03)	.08
West	0.77 (0.57-1.02)	.07

Abbreviations: AA, associate degree; GED, general equivalency diploma; NA, not applicable; NHIS, National Health Interview Survey.

^a^ Other race/ethnicity included Asian, American Indian/Alaska Native, and multiracial individuals.

Older individuals (HR, 1.02; 95% CI, 1.01-1.03), individuals diagnosed as having an SRC vs an NSRC (HR, 1.28; 95% CI, 1.06-1.54), and individuals with obesity (BMI ≥30) vs normal weight (BMI 18.5 to <25) (HR, 1.32; 95% CI, 1.06-1.63) had a higher probability of reporting a cigarette smoking cessation event after cancer diagnosis. Having a higher educational level was positively associated with the odds of quitting cigarette smoking after cancer diagnosis; individuals with an undergraduate degree (HR, 1.39; 95% CI, 1.08-1.79) or a postgraduate degree (HR, 1.61; 95% CI, 1.18-2.20) had a higher probability of reporting a cigarette smoking cessation event after cancer diagnosis compared with individuals with an educational level of high school graduate or GED. Cancer survivors living below the poverty level (HR, 0.62; 95% CI, 0.48-0.81) had a statistically significantly lower probability of reporting a cigarette smoking cessation event after cancer diagnosis compared with those living above the poverty level.

Using the survey Cox proportional hazards model, we found that the probability of having a cigarette smoking cessation event increased on average by 4.59% relative to the preceding year among US adult smokers diagnosed with cancer during 2000 to 2017. The value of the year of cancer diagnosis relative to a cigarette smoking cessation event is further shown in the Figure, with a detailed explanation given in the caption. For the convenience of health care professionals, we have developed a web app^18^ (eAppendix in the Supplement) to assist in calculating the probability of quitting cigarette smoking after cancer diagnosis based on the sociodemographic characteristics of patients.

**Figure.**
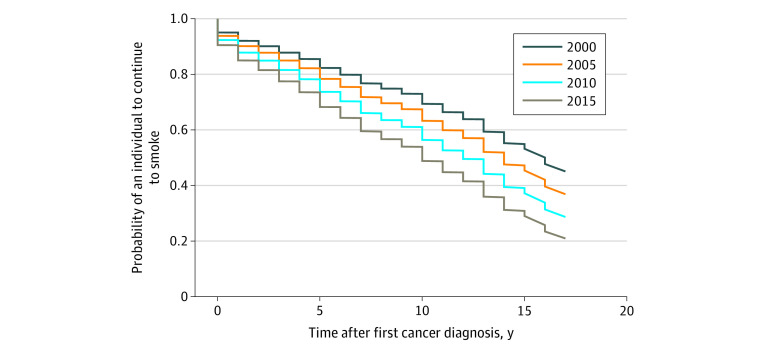
Hypothetical Survival Curves Showing the Prevalence of Cancer Survivors Who Were Smoking at the Time of First Cancer Diagnosis and Subsequently Continued to Smoke After Diagnosis of Cancer Using the Survey Cox Proportional Hazards Model Hypothetical curves are shown for individuals diagnosed with cancer in 2000, 2005, 2010, and 2015. To better explain these results, we take the example of four 50-year-old smokers diagnosed with non–smoking-related cancer but whose cancer was first diagnosed in different years (2000, 2005, 2010, and 2015) with the following characteristics: female, non-Hispanic White, educational level of high school graduate or general equivalency diploma, living above the poverty level, normal body mass index, no alcohol consumption, and residing in the Northeast US Census region. Our findings suggest that the woman who was diagnosed with cancer in 2015 has a higher probability of quitting cigarette smoking at a given time point after cancer diagnosis compared with the woman whose cancer was first diagnosed in 2010. Similarly, the latter woman has a higher probability of quitting cigarette smoking than the woman who was diagnosed with cancer in 2005, who in turn has a greater probability of quitting cigarette smoking than the woman who was first diagnosed with cancer in 2000. More precisely, the smoker who was diagnosed with cancer in 2000 has an 88.19% probability of continuing to smoke 5 years after cancer diagnosis compared with 85.45%, 82.13%, and 78.17% for the smokers who were first diagnosed with cancer in 2005, 2010, and 2015, respectively.

### Multiple Cancers and Decision to Quit

As an exploratory analysis, we also investigated the association between being diagnosed as having multiple cancers (n = 368) and the decision to quit smoking among cancer survivors who were smoking at the time of their first cancer diagnosis and continued to smoke until a second cancer diagnosis was made. We found 20.63% (95% CI, 15.24%-27.31%) and 22.70% (95% CI, 17.22%-29.30%) of cancer survivors who had a second cancer diagnosis quit within 1 year and 2 years after second diagnosis, respectively. We further investigated cigarette smoking cessation among those individuals who had a second cancer diagnosis within 1 year (n = 201) and 2 years (n = 250) of first cancer diagnosis. In individuals who reported a second cancer diagnosis within 1 year after first cancer diagnosis, 24.51% (95% CI, 16.46%-34.85%) quit within 1 year after second diagnosis, and 26.74% (95% CI, 18.62%-36.80%) quit within 2 years after second cancer diagnosis. In individuals who reported a second cancer diagnosis within 2 years of first cancer diagnosis, 23.60% (95% CI, 16.40%-32.71%) quit within 1 year after second diagnosis, and 26.03% (95% CI, 18.72%-34.95%) quit within 2 years after second diagnosis.

In comparison, quit rates after the first cancer diagnosis were 10.29% (95% CI, 8.60%-12.27%) within 1 year of cancer diagnosis and 12.33% (95% CI, 10.53%-14.39%) within 2 years of cancer diagnosis. Compared with cancer survivors who were smoking at the time of first cancer diagnosis, our findings suggest that individuals who were diagnosed as having a second cancer when still smoking after the first cancer diagnosis were more likely to quit smoking within 1 year and 2 years of second cancer diagnosis.

## Discussion

In this nationally representative sample of the US adult population of cancer survivors, increasing trends in cigarette smoking cessation were observed with the year of cancer diagnosis from 2000 to 2017. However, the pace of cigarette smoking cessation rates in this high-risk population was slow at less than 5% increment relative to the cessation rate in the preceding year. Over the study period, almost one-quarter of cancer survivors were current smokers at the time of cancer diagnosis. Despite some progress, these persistently high rates of smoking among cancer survivors may adversely alter their quality of life, survival, and disease prognosis.^2^ High rates of continuing smoking have also been observed in another study,^19^ in which only one-third of the cancer survivors who were currently smoking intended to quit smoking, an antecedent to quit attempts and eventual cessation.

The increasing trends in cigarette smoking cessation among cancer survivors found in this study are consistent with declining trends in cigarette smoking observed in the US general population over the last decades. Indeed, current smoking among US adults declined from 20.9% in 2005 to 15.5% in 2016 and to 13.7% in 2018, and the proportion of ever smokers who have quit has increased.^20^ The percentage who have quit increased from 50.8% in 2005 to 59.0% in 2016 among those who have ever used cigarettes.^20^ This notable public health achievement may be explained by the nationwide implementation of population-based tobacco control interventions, including high-impact tobacco education campaigns like the Centers for Disease Control and Prevention’s Tips From Former Smokers campaign^21^ and the US Food and Drug Administration’s Every Try Counts campaign,^22^ coupled with barrier-free access to evidence-based cessation treatments. In an analysis of the changes in cigarette smoking cessation counseling among US adult cigarette smokers, the proportion who reported receiving physician advice to quit smoking has increased.^5^ Cancer diagnosis represents a teachable moment^3,4,23^: advice from a clinician about the health benefits of quitting can promote cigarette smoking cessation. Despite this progress at the population level, smoking prevalence among cancer survivors remains high. In a recent analysis of the 2017 NHIS data, 1 in 7 cancer survivors in the United States reported being a current cigarette smoker.^7^ Therefore, coordinated action at cancer diagnosis and treatment centers and hospitals, at survivorship clinics, and at the level of primary health care professionals is needed to continue progress toward reducing tobacco-related disease and death among cancer survivors in the United States. Considering that a high proportion of US adult cancer survivors continue to smoke, integration of behavioral and pharmacological cigarette smoking cessation interventions into oncology care is imperative to improve quit rates in this high-risk population.^24,25,26^

In the survey Cox proportional hazards model, age, educational level, poverty level, and BMI were associated with statistically significantly altered cigarette smoking cessation after cancer diagnosis, indicating socioeconomic disparities in cessation among cancer survivors. For this reason, we did not rely on crude prevalence to assess temporal trends in quitting cigarette smoking among cancer survivors; rather, we used the prevalence adjusted on these potential confounders per the survey Cox proportional hazards model. Such sociodemographic and economic disparities are observed in the US general population, suggesting that cigarette smoking is not declining at the same rate in these vulnerable groups. For instance, cigarette smoking in 2018 was especially high among the following groups: men, those aged 25 to 64 years, those who had a lower educational level, those living below the poverty level, and those who lived in the Midwest or South, as well as individuals who had serious psychological distress and individuals who were uninsured or underinsured.^20^ Therefore, addressing these disparities with evidence-based interventions is critical to improve the overall cigarette smoking cessation rate among cancer survivors and in the US population.

### Limitations

This study has some limitations. Because of the cross-sectional nature of the data, our results could have been altered by survival bias because smokers who receive a cancer diagnosis were less likely to survive and thus to participate in the study. Because information about treatment was not collected in the NHIS, we could not distinguish between cancer survivors who were still under treatment and those who had already completed their treatment course and further survived free of cancer. When cancer diagnosis and cigarette smoking cessation occurred in the same year, it was not possible to determine the sequence of occurrence of these 2 events. Therefore, we excluded these individuals from our study, which may have resulted in an underestimation of the proportion of smokers who quit smoking after cancer diagnosis. The number of cancer survivors who were smokers at the time of cancer diagnosis for 2016 and 2017 was derived from only 1 or 2 survey cycles (2017 and/or 2018), resulting in a smaller sample size in these years. Therefore, the results from recent years should be interpreted more cautiously. Finally, this study used self-reported data; however, self-reported smoking status has been shown to correlate with serum cotinine levels.^27^

## Conclusions

In this nationally representative survey of the US adult population, the likelihood of cigarette smoking cessation among cancer survivors increased with the year of cancer diagnosis from 2000 to 2017; however, the improvement is incremental, and the prevalence of smoking remained high among this population. Urgent action is needed to improve cigarette smoking cessation rates in this high-risk population. Cancer care physicians and other specialists and clinicians should reinforce cigarette smoking cessation efforts among their patients. In addition, greater emphasis should be placed on implementing targeted cessation programs in oncology centers, such as the National Cancer Institute’s Moonshot Program,^28^ to help patients with cancer stop smoking.

## Notes

Supplement. **eAppendix.** Software to Compute the Probability of Quitting Smoking After a First Cancer Diagnosis
