# Supplementary material for: Assessment of Trends in Cigarette Smoking Cessation After Cancer Diagnosis Among US Adults, 2000 to 2017
Source: JAMA Netw Open. 2020 Aug 3;3(8):e2012164. doi: 10.1001/jamanetworkopen.2020.12164 (PMC7399749; doi:10.1001/jamanetworkopen.2020.12164)
Supplement: Supplement. — eAppendix. Software to Compute the Probability of Quitting Smoking After a First Cancer Diagnosis [file jamanetwopen-3-e2012164-s001.pdf]

Supplementary Online Content

Talluri R, Fokom Domgue J, Gritz ER, Shete S. Assessment of trends in cigarette smoking cessation after cancer diagnosis among US adults, 2000 to 2017. *JAMA Netw Open*. 2020;3(8):e2012164. doi:10.1001/jamanetworkopen.2020.12164

eAppendix. Software to Compute the Probability of Quitting Smoking After a First Cancer Diagnosis

This supplementary material has been provided by the authors to give readers additional information about their work.

© 2020 Talluri R et al. *JAMA Network Open*.

eAppendix. Software to Compute the Probability of Quitting Smoking After a First Cancer Diagnosis

The software is available at http://rtalluri.shinyapps.io/smokcess/

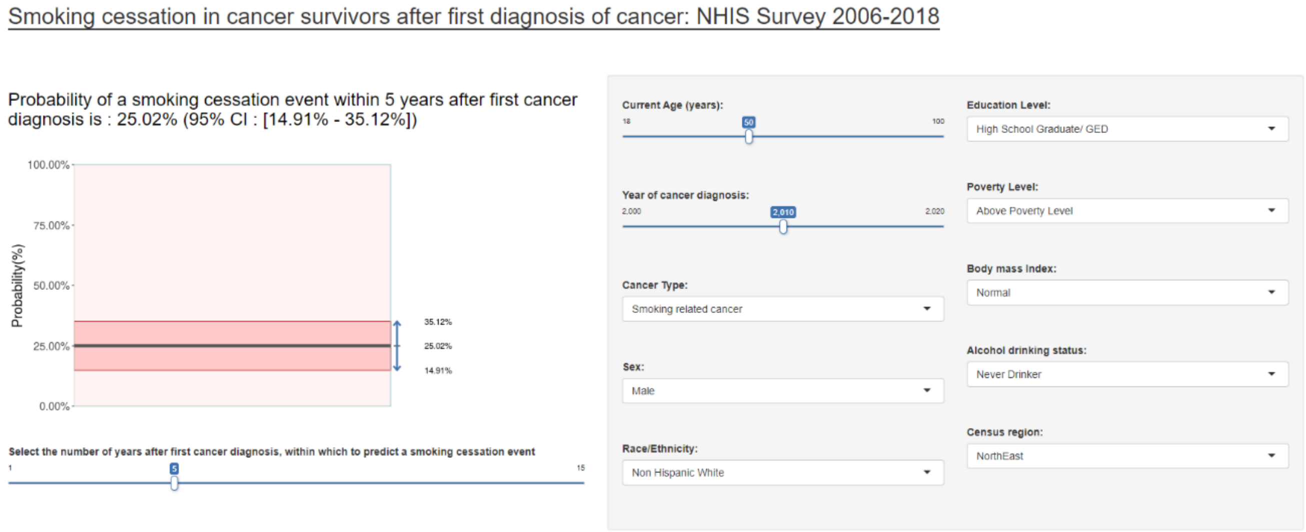

Usage

Output: This software program outputs the predicted probability and its 95% confidence interval, of a cancer survivor (who was a smoker at the time of diagnosis) to quit smoking within a specified time interval based on the 2006-2018 NHIS survey data.

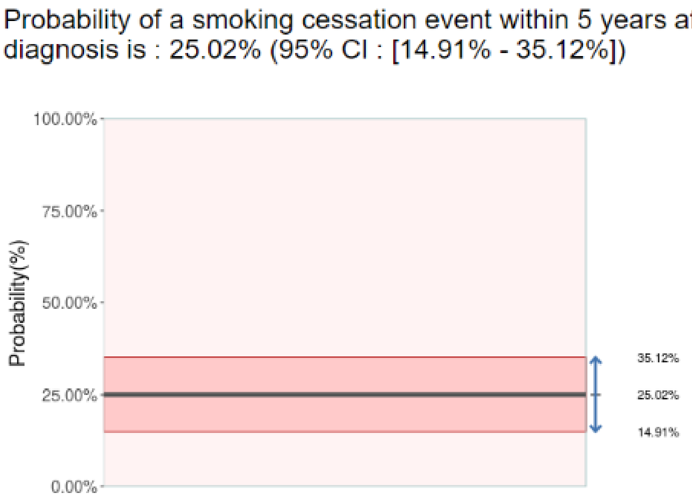

© 2020 Talluri R et al. *JAMA Network Open*.

Input:

1. The specified time interval within which the probability of smoking cessation is to be computed.

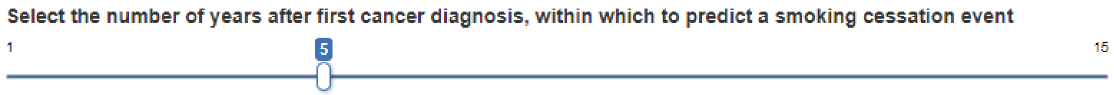

2. Social, economic, and demographic characteristics of the individual:

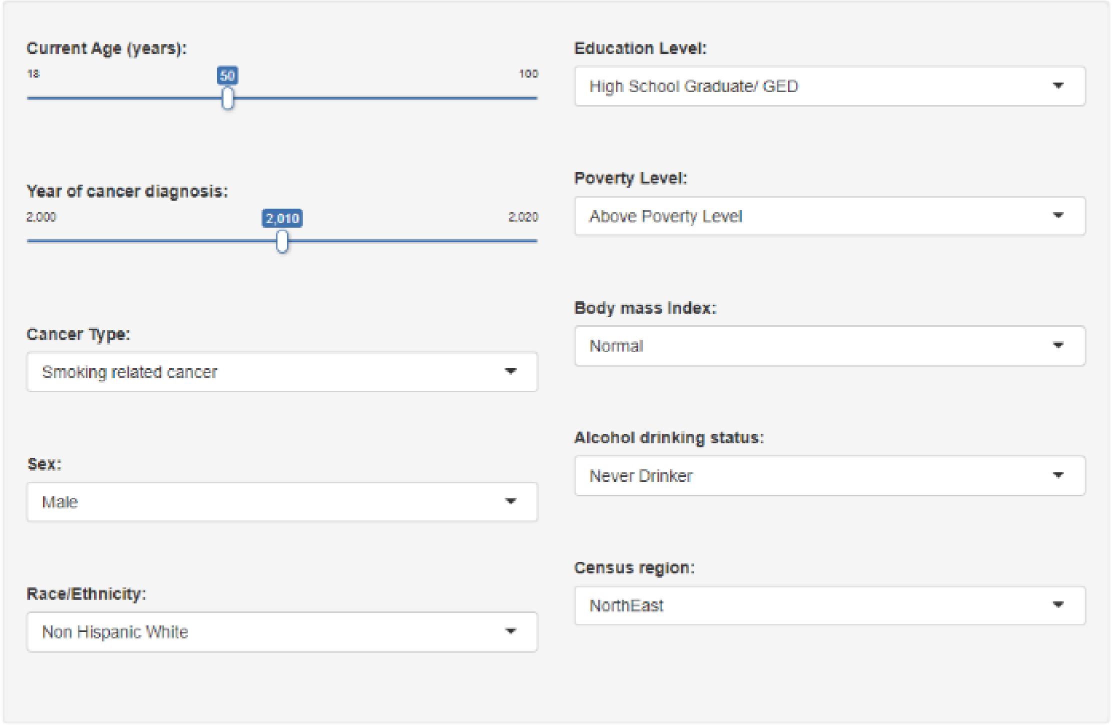

© 2020 Talluri R et al. *JAMA Network Open*.
